# Weight gain a risk factor for mortality in patients with acute kidney injury requiring continuous renal replacement therapy

**DOI:** 10.1186/2197-425X-3-S1-A466

**Published:** 2015-10-01

**Authors:** C Maldonado Toral, L Dono, A Parra, N Ramos, R Albertos, E Papiol, M Pérez

**Affiliations:** Hospital Universitari Vall d'Hebron, Critical Care, Barcelona, Spain; Hospital Universitari Vall d'Hebron, Nephrology Barcelona, Spain

## Introduction

Renal failure is one of the most common complications in critically ill patients, with a progressive increase in the need of renal replacement therapy (RRT). As well-know the timing is pretty related with the improvement and outcome. But also the treatment dose and filter lifespan are variables that interfere in the results.

## Objectives

Assess the prescribed treatment and the difference with the treatment achieved. Also evaluate the variation on vasoactive drugs and the weight impact on mortality rate.

## Methods

A retrospective descriptive study was performed during 14 months, between January 2014 and February 2015. All patients required Intensive Care Unit (ICU) admission and RRT regardless of the pathology that led them in to the ICU. Demographic, clinical and biochemical data was recorded at the day of the admission and at the beginning of RRT. Data are reported as frequency (percentage) and median (interquartile range). Statistical test used were Pearson's test or Fisher test, U de Mann-Whitney test and logistic regression.

## Results

One hundred eighty nine patients were admitted in ICU during study period, sixty one of them required RRT, 64.5% male, median age was 64(54-60) years and body mass index 28(23-34). At the ICU admission, APACHE II and SOFA score were 34(26-38) and 11(9-13), respectively. Renal failure etiology was multifactorial cause 54.8%, septic 29% and ischemia 4.9%. Most of patients required mechanical ventilation (84.7%) and vasoactive drugs (91.8%).

More than half of RRT were initiated in the first 24h. Treatment dose prescribed during first three days of RRT were 32(25-40) ml/kg/h, 32(25-35) ml/kg/h and 25(20-34) ml/kg/h. However treatment actually achieved was a 4% less. At the beginning of RRT, median vasoactive drugs (noradrenaline) was 0.7(0.17-1.35) mcg/kg/min and median creatinine 2.38(1.57-3.69) mg/dL, decreasing 48h later to 0.37(0.15-0.76) mcg/kg/min and 1.50(1.11-1.96) mg/dL.

Median ICU LOS was 12(4-29) days with median RRT 4(1-6) days and overall mortality rate of 63.3%. An increase more than 5% in weight during the first 48 hours of RRT was associated with increased mortality. In addition, SOFA score showed to be a good predictor of mortality at ICU admission and during the first 48 hours of RRT (p < 0.01).

## Conclusions

Patients with RRT present a high mortality. A weight gain during first 48h of RRT has been related to increase mortality rate. Furthermore it has been shown a reduction in the vasoactive drugs.

## References

[1] Bagshaw S, Uchino S, Kellum JA, Morimatsu H, Morgera S, Schetz M (2013). Association between renal replacement therapy in critically ill patients with severe acute kidney injury and mortality. J Crit Care.

[2] Fülöp T, Pathak MB, Schmidt DW, Lengvárszky Z, Juncos JP, Lebrun CJ (2010). Volume-Related Weight Gain and Subsequent Mortality in Acute Renal Failure Patients Treated with Continuous Renal Replacement Therapy. ASAIO J.

